# Erythropoietin Ameliorates Ischemia/Reperfusion-Induced Acute Kidney Injury via Inflammasome Suppression in Mice

**DOI:** 10.3390/ijms21103453

**Published:** 2020-05-13

**Authors:** Jihye Kwak, Jin Hyun Kim, Ha Nee Jang, Myeong Hee Jung, Hyun Seop Cho, Se-Ho Chang, Hyun-Jung Kim

**Affiliations:** 1 Division of Nephrology, Department of Internal Medicine, College of Medicine, Gyeongsang National University and Gyeongsang National University Hospital, Jinju 52727, Korea; jihye7503@gmail.com (J.K.); asaku@naver.com (H.N.J.); mars36@daum.net (H.S.C.); shchang@gnu.ac.kr (S.-H.C.); 2 Biomedical Research Institute, Gyeongsang National University Hospital, Jinju 52727, Korea; ajini7044@hanmail.net (J.H.K.); yallang7@hanmail.net (M.H.J.); 3 Institute of Health Sciences, Gyeongsang National University, Jinju 52727, Korea

**Keywords:** acute kidney injury, erythropoietin, inflammasome, ischemia/reperfusion injury

## Abstract

Acute kidney injury (AKI) is the most common condition in hospitalized patients. As ischemia/reperfusion-induced AKI (IR-AKI) is as a major contributor to end-stage disease, an effective therapeutic intervention for IR-AKI is imperative. Erythropoietin (EPO) is a potent stimulator of erythroid progenitor cells and is significantly upregulated during hypoxia. Here, we investigated the renoprotective effects of EPO in an IR-AKI mouse model. Mice were assigned to sham, EPO only, and IR only groups, and the IR group was treated with EPO prior to injury. EPO was administered twice at 30 min prior to bilateral renal artery occlusion, and 5 min before reperfusion, with all mice sacrificed 24 h after IR-AKI. The serum was harvested for renal functional measurements. The kidneys were subjected to histological evaluation, and the biochemical changes associated with renal injury were assessed. EPO significantly attenuated the renal dysfunction associated with IR-AKI, as well as tissue injury. Apoptotic cell death and oxidative stress were significantly reduced in EPO-treated mice. Macrophage infiltration and expression of ICAM-1 and MCP-1 were also significantly reduced in EPO-treated mice. Furthermore, the expression of inflammasome-related factors (NLRP1, NLRP3, and caspase-1 cleavage), via the activation of the COX-2 and NF-κB signaling pathways were significantly reduced following EPO treatment. To our knowledge, this is the first study to demonstrate that inflammasome-mediated inflammation might be a potential target of EPO as a treatment for ischemic AKI.

## 1. Introduction

Acute kidney injury (AKI) is the most common condition in hospitalized patients [1]. As ischemia/reperfusion-induced AKI (IR-AKI) is as a major contributor to end-stage disease, an effective therapeutic intervention for IR-AKI is imperative.

Renal inflammation is a universal response to both infectious and noninfectious insults. Experimental models suggest that pathogen-associated molecular patterns (PAMPs), and the uncontrolled release of danger-associated molecular patterns (DAMPs) from damaged or dying cells drive inflammatory responses, and subsequent tissue and organ injury. Inflammasomes are multiprotein complexes that regulate cytokine maturation, inflammation, and cell death via the activation of certain caspases [2]. Five receptors are known to assemble inflammasomes, which are the nucleotide-binding oligomerization domain (NOD)-like receptor (NLR) proteins: NLRP1, NLRP3, and NLRC4; as well as the absent in melanoma 2 (AIM2)-like receptors, AIM2 and pyrin [3]. The NLRP3 inflammasome is a cytosolic complex consisting of NLRP3, ASC, and caspase-5. BID is a pro-apoptotic inflammasome-related protein. NLRP3 is the most well-studied of the inflammasome-related proteins and is activated by DAMPs, which regulate the secretion of pro-inflammatory cytokines such as IL-1β and IL-18. These inflammasome components have been directly implicated in renal inflammation injury [4].

Erythropoietin (EPO) is a potent growth factor of erythroblasts that is significantly upregulated during hypoxia. Recombinant human EPO, first developed in 1989, is one of the most important factors in the treatment of patients with chronic kidney disease. Treatment with erythropoiesis-stimulating agents has led to significant improvements in patients’ quality of life [5,6]. Furthermore, the anti-inflammatory and antioxidant effects of EPO in AKI have been demonstrated in renal cell and animal models [7]. Here, we investigated the renoprotective mechanisms of EPO in IR-AKI mice, as well as the role of the inflammasome in mediating these effects.

## 2. Results

### 2.1. EPO Ameliorates IR-Induced Renal Dysfunction and Tissue Damage

**Figure 1:**
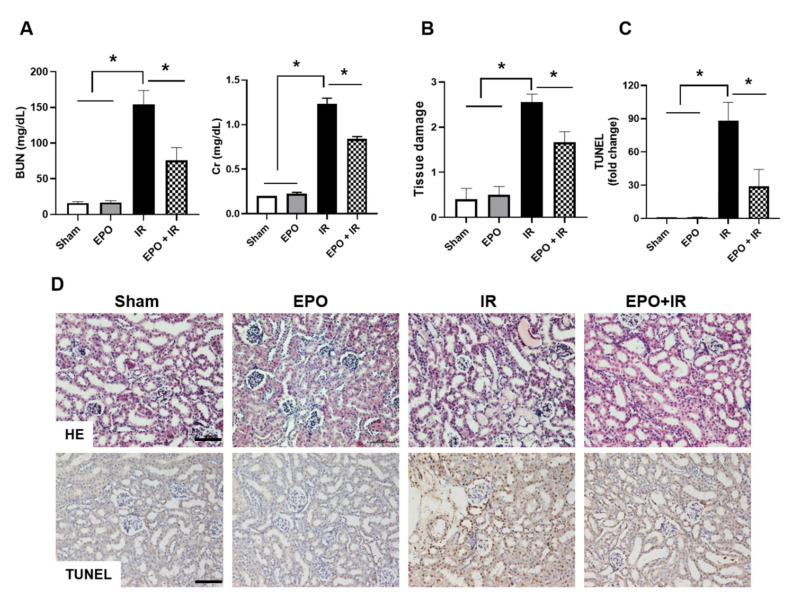
Effects of Erythropoietin (EPO) administration on renal function and morphological changes in IR-AKI. EPO (500 IU/kg body weight) was administered into the tail vein. Mice were sacrificed 24 h after ischemic injury for blood and kidney sampling. The serum blood urea nitrogen (BUN) and serum creatinine (Cr) levels were measured (**A**), and histological changes and renal apoptosis were examined by H&E staining and TUNEL assay, respectively (**B–D**). Tissue damage was quantified as described in the Materials and Methods section (**B**). Quantitative analysis of TUNEL-positive cells was performed (**C**). Scale bar, 100 μm. Data are means ± SEM. *p < 0.05.

Serum blood urea nitrogen (BUN) and serum creatinine (Cr) levels were markedly elevated in the IR group, and pre-treatment with EPO significantly attenuated BUN and Cr elevation in IR mice (Figure 1A). To confirm IR-induced tissue injury, hematoxylin and eosin (H&E) staining was performed. Kidneys from the IR group showed extensive tubular injury, characterized by tubular atrophy, cast formation, and loss of brush border. These pathohistological changes were significantly attenuated in the IR group treated with EPO. The sham and EPO groups exhibited no changes in renal morphology (Figure 1B,D). Apoptosis-mediated tubular injury is implicated in IR-induced AKI [8]. EPO significantly decreased IR-induced apoptosis in tubular epithelial cells, as reflected by TUNEL-positive signals (Figure 1C,D).

### 2.2. EPO Administration Significantly Attenuates Inflammatory Cell Infiltration

**Figure 2:**
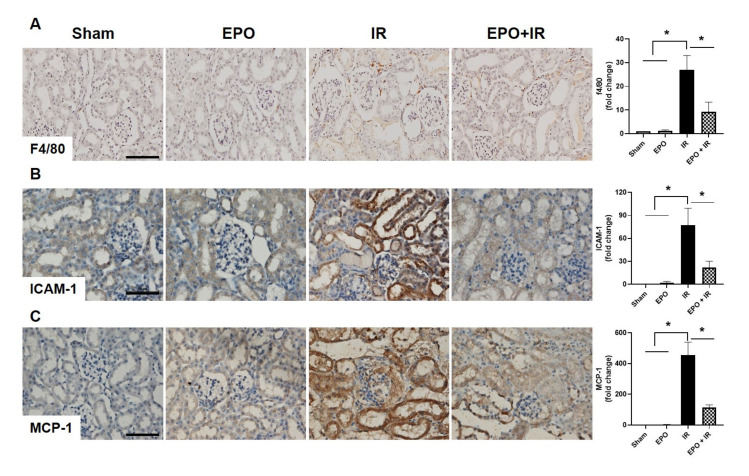
Effects of EPO on macrophage infiltration after IR injury. To verify macrophage infiltration, immunohistochemical staining of F4/80 was performed (**A**). F4/80-positive signals were found in the interstitial areas of kidneys after IR. Immunohistochemical staining of the inflammatory mediators ICAM-1 (**B**), and MCP-1 (**C**), was also examined. Each signal was analyzed by densitometry. Scale bar, 50 μm. Data are means ± SEM. *p < 0.05.

Macrophage infiltration is a well-defined feature of tissue inflammation in IR-AKI [9]. Intracellular adhesion molecule-1 (ICAM-1) and macrophages/monocytes chemotactic protein-1 (MCP-1), are the common inflammation-involved factors in AKI, including ischemic AKI [10], and are associated with the infiltration of macrophage in ischemic AKI [11]. EPO administration significantly decreased macrophage infiltration in the EPO+IR group compared to the IR only group (Figure 2A). The expression levels of ICAM-1 and MCP-1 were also significantly reduced in the EPO+IR group (Figure 2B,C).

### 2.3. EPO Reduces Oxidative Stress and NF-κB Pathway Activation

**Figure 3:**
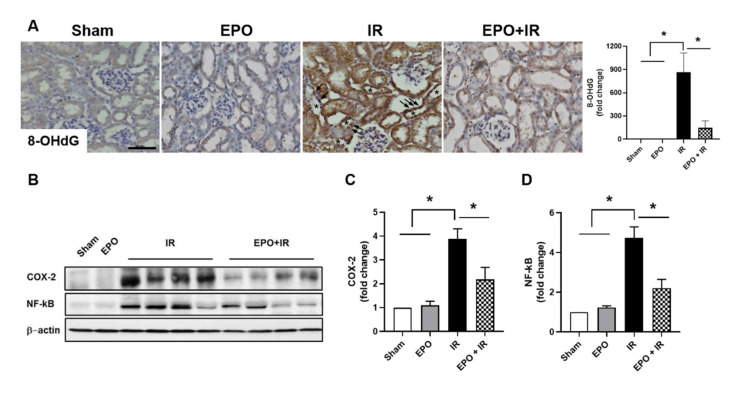
EPO decreases IR-induced oxidative stress and NF-κB pathway activation. Sections were stained with anti-8-OHdG as a marker for oxidative stress. Signals were analyzed by densitometry (**A**). Kidney extract was prepared 24 h after IR injury. COX-2 and NF-κB protein expression were analyzed by Western blot (**B**). The histograms show the results of densitometric analysis of bands normalized to β-actin (**C**,**D**). Scale bar, 50 μm. Data are presented as mean ± SEM. *p < 0.05.

Oxidative stress induced by damaged tissues, as well as the migration of inflammatory cells into these tissues, is a potent activator of the nuclear factor kappa B (NF-κB) signaling pathway, and a major driver of pathologic inflammation [12]. Immunohistochemical staining of 8-OHdG, a reactive oxygen species (ROS)-induced DNA damage marker, was performed to investigate the effect of EPO on IR-induced oxidative stress in the kidney. 8-OHdG-positive signals were detected in the nuclei of tubular epithelial cells in the IR only group (arrow in Figure 3A), and these signals were significantly decreased by EPO treatment (Figure 3A). We also examined the activation of the NF-κB signaling pathway and COX-2 expression as a target of NF-κB signaling. Marked induction of COX-2 and p-NF-κB protein expression was detected in the IR kidney tissues. EPO reduced the expression of these proteins (Figure 3B–D).

### 2.4. EPO Decreased Inflammasome Activation

**Figure 4:**
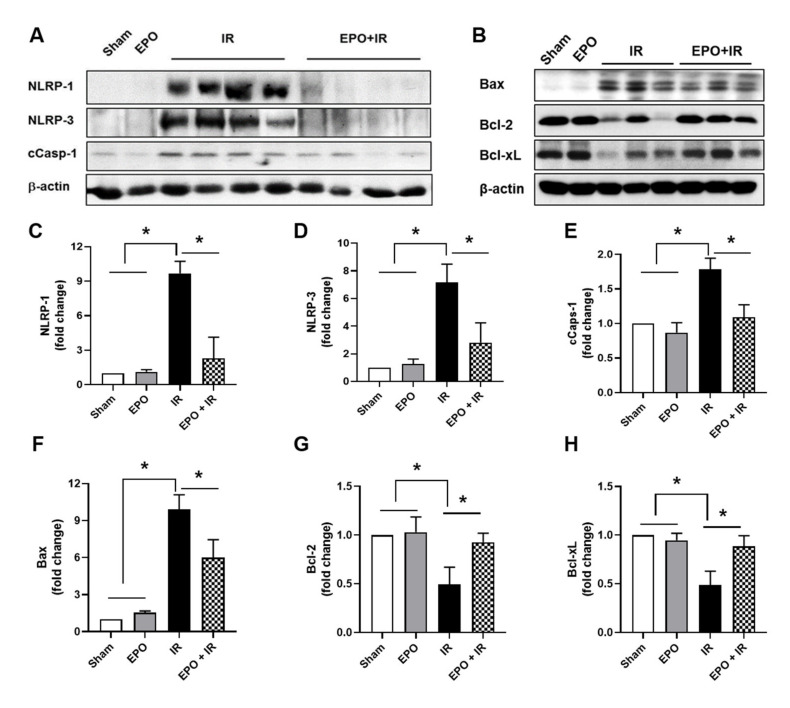
EPO decreases inflammasome expression and changes mitochondrial apoptotic effectors expression. Renal extract was prepared 24 h after ischemia/reperfusion injury (IRI). Expression levels of NLRP-1, NLRP-3, and cleaved caspase-1 (**A**) and Bax, Bcl-2, and Bcl-xL (**B**) were analyzed by Western blot. Quantitative analysis of NLRP-1 (**C**), NLRP-3 (**D**), cleaved caspase-1 (**E**), Bax (**F**), Bcl-2 (**G**), and Bcl-xL (**H**) were performed, with results normalized to β-actin. Data are presented as mean ± SEM. *p < 0.05.

Next, we examined the expression of inflammasome-related factors. The NLRP3 inflammasome is an important mediator of ischemic AKI [13,14]. Significant increases in NLRP-1 and NLRP-3 expression were observed in the kidneys of IR mice, and these increases were significantly attenuated in the IR+EPO group (Figure 4A,C,D). Cleaved caspase-1 expression was also ameliorated by EPO administration (Figure 4A,E). Vince et al. demonstrated that mitochondrial apoptotic effectors trigger NLRP3 inflammasome [15]. We investigated the expression levels of the mitochondrial apoptotic machinery-related factors such as Bax, Bcl-2, and Bcl-xL (Figure 4B,F–H). The expression of Bax, a mitochondrial pro-apoptotic effector, was increased, whereas Bcl-2 and Bcl-xL, mitochondrial anti-apoptotic effectors, were decreased in the IR only group. However, these expression levels were reversed in the EPO + IR group.

## 3. Discussion

This study showed that EPO protected against IR-AKI via the inactivation of inflammasome-dependent signaling pathways, as well as the inhibition of oxidative stress. EPO not only protected against IR-induced histologic and biochemical changes, but also against the loss of renal function.

EPO has shown protective effects in several experimental AKI models, through various mechanisms including the regulation of microvascular injury [7], the reduction in tubulointerstitial injury (independent of its hemopoietic effects) [16], anti-inflammatory and anti-apoptotic effects [17], decreased fibrocyte accumulation [18], and the modulation of macrophage polarization [19,20]. Notably, EPO has shown efficacy in animal models of IR-AKI [21], as well as in an in vitro hypoxia-reoxygenation study [22], via the regulation of PI3K/Akt signaling.

The cellular targets of EPO include NF-κB, COX-2, and mitogen-activated protein kinase (MAPK). EPO was shown to prevent sepsis-related AKI in a rat model by inhibiting NF-κB and upregulating endothelial nitric oxide synthase (eNOS) [23]. Similarly, recombinant human EPOsuppressed activity in the NF-κB and inducible nitric oxide synthase (iNOS) pathways in a rhabdomyolysis-AKI rat model [20]. Additional anti-apoptotic effects of EPO were shown to be mediated by the NF-κB pathway in an IR mouse model [24]. Finally, the inhibition of COX-2 ameliorated IR-AKI in both rats [25] and mice [26]. In this study, EPO administration ameliorated renal apoptosis, NF-κB activation, and COX-2 expression by IR. Our data suggest that EPO decreases IR-induced renal apoptosis via the regulation of the NF-κB pathway.

In AKI, macrophages exacerbate the inflammatory response, as well as the associated cytotoxic effects, via the generation of ROS and proinflammatory cytokines [27]. ROS, released by damaged tissues and inflammatory cells, are a potent inducer of NF-κB activation [12]. This activation of NF-κB plays an important role in disease progression, by promoting the synthesis of inflammatory mediators, leading to the transcription of adhesion molecules such as ICAM-1 and chemotactic factors such as MCP-1 [28]. The inhibition of ICAM-1 expression results in decreased leukocyte adhesion and renal inflammation in an IR-AKI model [29]. This study showed that EPO administration decreased renal oxidative stress, which may have resulted from a reduction in macrophage infiltration. Furthermore, EPO ameliorated the levels of other inflammatory mediators, such as ICAM-1 and MCP-1. This could be due to the inactivation of NF-κB because of decreased oxidative stress.

The NLRP3 inflammasome is activated in both acute and chronic kidney disease. The inhibition of NLRP3, via NLRP3 inflammasome knockout or cathepsin-mediated NLRP3 inhibition, has been shown to confer significant protection against IR-AKI in mice [9,13]. Similarly, caspase-1, a downstream target of NLRP3, has also been shown to play an important role in IR-AKI [30]. IR-induced activation of the NLRP3 inflammasome results in prolonged caspase-1 cleavage [14]. Although the NLRP3 inflammasome is an important mechanism in IR-AKI, and candidates for blocking NLRP3 inflammasome activation such as hydroxychloroquine are being developed for IR-AKI [9], no studies have examined whether EPO can inhibit activation of the NLRP3 inflammasome in IR-AKI. Our results showed that EPO-mediated protection against IR-AKI was associated with significant decreases in caspase-1 cleavage, as well as in NLRP1 and NLRP3 inflammasome activation. This suggests that the EPO-induced suppression of caspase-1 cleavage via the inflammasome has potential as a mechanism of renal protection after IR injury. Recently, it has been reported that the mitochondrial apoptotic effectors trigger NLRP3 inflammasome through caspase-3 and -7 activation [15]. They showed that in macrophage, BAX/BAK, the mitochondrial apoptotic effectors, activate caspase-3 and 7 and activated caspase-3 and 7 caused potassium ion efflux and ultimately, triggered NLRP3 inflammasome formation. We examined if the mitochondrial apoptotic machinery could be another cellular target of EPO on suppression of inflammasome activation in IR-AKI. EPO ameliorated the expression of Bax and preserved the expression of Bcl-2 and Bcl-xL. Thus, these data suggest that EPO protects against IR-AKI by inhibition of the mitochondrial apoptotic effectors to trigger inflammasome.

As mentioned above, various events such as apoptosis, inflammation, hypoxic injury, and oxidative stress by production of reactive oxygen species, are involved in the pathogenesis of IR-AKI [31,32,33]. It is advantageous to inhibit these events in the prevention and treatment of IR-AKI. In light of our data in this study, EPO protects against IR-AKI in two ways. Firstly, EPO decreases macrophage-involved inflammation as follows. IR-AKI begins from macrophage infiltration. Infiltrated macrophage generates ROS and produces proinflammatory cytokines in damaged tissues. ROS and proinflammatory cytokines activate the NF-κB signaling pathway that promotes the transcription of adhesion molecules like ICAM-1 or MCP-1. These adhesion molecules facilitate the macrophage migration again. Ultimately, tubular epithelial cells are in apoptotic cell death and kidney dysfunction is incurred. In this study, although we cannot explain exactly which steps are first for EPO working, we cannot rule out the reduced macrophage infiltration. Secondly, NLRP3 among inflammasome components is well known to various kidney disease including IR-AKI. The protective effect of EPO on AKI is very well-described; however, it is not for inflammasome, even reported in acute lung injury [34]. According to our data, EPO significantly decreased the expression levels of the inflammasome-related factors NLRP1, NLRP3, and cleaved caspase-1. Moreover, EPO also reduced the expression of the mitochondiral apoptotic effects to trigger NLRP3 inflammasome.

The pathogenesis of IR-AKI contains multiple complex steps and mechanisms. Therefore, targetting a single step and a single mechanism is not helpful to treat IR-AKI. To our knowledge, this is the first report to indicate that EPO might be a potential option for multiple targets including the suppression of the inflammasome-mediated inflammation as a prevention and treatment measure for ischemic AKI.

## 4. Materials and Methods

### 4.1. Ethics Statement

This study was approved on 16 February 2016 by the Gyeongsang National University Institutional Animal Care and Gyeongsang National University Institutional Ethics Committee (GNU160216-M0009).

### 4.2. Ischemia/Reperfusion-Induced AKI

Male C57BL/6 mice (10 weeks of age) were maintained in a 12-h light/dark cycle in a temperature- and humidity-controlled facility. Standard mice chow and water was provided ad libitum. Mice were assigned to sham, EPO only, IR only, and EPO administered prior to IR groups. EPO (500 unit/kg; EPOKINE^®^, Erythropoietin-α, CJ Healthcare) was administered twice at 30 min prior to bilateral renal artery occlusion and 5 min before reperfusion and the mice were then sacrificed at 24 h after IR-AKI. Mice were anesthetized with IP Avertin (2,2,2-tribromoethanol, Sigma-Aldrich, St. Louis, MO, USA). The renal pedicles were bilaterally clamped for 40 min with microaneurysm clamps after a midline incision. The time of ischemia was chosen to obtain a reversible model of ischemic AKI and avoid animal mortality. After clamp removal, kidneys were observed for restoration of blood flow by the return to their original color. The abdomen is closed in two layers. Sham surgery consisted of the same surgical procedure except that clamps were not applied. During the first 24 h of the reperfusion period, the animals were kept in an incubator at 29 °C. Animals were sacrificed at 24 h after ischemia. Blood and kidney tissues were harvested. All experiments were performed in triplicate with *n* = 7 animals in each group.

### 4.3. Histopathology

Tissues were fixed in 4% paraformaldehyde in 0.1 M PBS, embedded in paraffin, and cut into 5-μm. The sections were stained with H&E. The semi-quantitative scoring for H&E staining was examined on the degree of interstitial injury that assigned points (0 to 3) for the extent of interstitial fibrosis and tubular atrophy (defined as luminal dilation, loss of brush border and flattened tubular epithelial cells). Tissue injury was scored by grading the percentage of affected under a high-powered field (×400): 0, 0%; 1, <30%; 2, 31% to 60%; 3, 61% to 100%. All scorings were summed and represented as average values on a graph, and signals were analyzed using NIS-Elements BR 3.2 (Nikon, Tokyo, Japan).

### 4.4. TUNEL Assay

The degree of apoptosis was assessed using a TUNEL assay. Detection of DNA fragmentation was performed using a kit from Roche Applied Sciences (Indianapolis, IN, USA). A semiquantitative analysis was performed by counting the number of TUNEL-positive cells per field, in the renal tissue, at ×400 magnification. At least 10 areas in the cortex per slide were randomly selected. The mean number of brown colored cells in these selected fields was expressed as the density of TUNEL-positive cells.

### 4.5. Immunoblotting

The samples were obtained from the kidneys for immunoblotting. The tissues were homogenized in RIPA buffer (#89900. Thermo scientific. Waltham, MA, USA). Amounts of protein were measured using the BCA assay kit (Pierce, Rockford, IL, USA), according to the manufacturer’s protocol. Proteins (50 µg) were loaded and electroblotted. The blots were probed with primary antibodies against monoclonal anti-caspase-1 (Abcam) and polyclonal anti-NLRP1 (Cell signaling, Danvers, MA, USA), NLRP-3 (Abcam, Cambridge, UK), COX-2 (Cell signaling), and NF-κB (Santa Cruz Biotechnology, Santa Cruz, CA, USA) at 4 °C overnight. The primary antibody was visualized by a secondary antibody and an ECL kit (Amersham Pharmacia Biotech, Piscataway, NJ, USA). The β-actin antibody (Sigma, St. Louis, MO, USA) served as the loading control. The densitometric analysis was performed for quantitative analysis of all data.

### 4.6. Immunohistochemistry

After deparaffinization, the sections were incubated with primary antibodies against monoclonal anti-ICAM-1 (BD Bioscience, Franklin Lakes, NJ, USA), MCP-1 and polyclonal anti-F4/80 (Santa Cruz), MCP-1 (Santa Cruz), 8-OHdG (Abcam, Cambridge, UK), followed by biotin-conjugated secondary IgG (diluted 1:200; Vector Laboratories, Burlingame, CA, USA), avidin–biotin–peroxidase complex (ABC Elite Kit; Vector Laboratories), and DAB. Next, we visualized the sections by light microscopy and captured and analyzed digital images using NIS-Elements BR 3.2 (Nikon, Japan).

### 4.7. Statistical Analysis

Statistical analyses were performed using GraphPad Prism software (version 8.0; GraphPad Software Inc., La Jolla, CA, USA). Data were evaluated using one-way ANOVA with Tukey’s multiple comparison test (for comparison all groups). All statistical testes used *p* < 0.05 to indicate significance.

## Abbreviations

AKI	Acute kidney injury
BUN	Blood urea nitrogen
EPO	Erythropoietin
Cr	Serum creatinine
eNOS	Endothelial nitric oxide synthase
ICAM-1	Intracellular adhesion molecule-1
iNOS	Inducible nitric oxide synthase
IRI	Ischemia/reperfusion injury
MCP-1	Monocytes chemotactic protein-1
NF-κB	Nuclear factor kappa B
NLRP	Nucleotide-binding oligomerization domain (NOD)-like receptor (NLR) proteins
ROS	Reactive oxygen species
